# Surface Plasmon Resonance as a Tool in Antiviral Drug Discovery Research

**DOI:** 10.3390/bios16030136

**Published:** 2026-02-26

**Authors:** Katarzyna E. Wegrzyn, John M. Matsoukas

**Affiliations:** 1 Laboratory of Molecular Biology, Intercollegiate Faculty of Biotechnology of the University of Gdansk and the Medical University of Gdansk, University of Gdansk, Abrahama 58, 80-307 Gdansk, Poland; 2 Department of Chemistry, University of Patras, 26504 Patras, Greece

**Keywords:** sensor chip, immobilization, viral entry, host receptor, small-molecule inhibitor

## Abstract

Viruses are an indispensable part of the environment we live in. The occurrence of seasonal and pandemic infections underscores the urgent need to develop new antiviral drugs or repurpose existing ones. Among the methods used in research on new antiviral molecules, surface plasmon resonance (SPR) has a well-established position due to its diverse applications in interaction analysis. It can be used to investigate various molecules (proteins, nucleic acids, small-molecular drugs) in different configurations and in real time. Although it is a gold-standard method for biomolecular interaction analysis, it is not free of constraints. Here, we review research on SPR in antiviral drug discovery. We focus on experimental design and discuss the application of SPR to investigate key stages of viral infection and to characterize antiviral interactions. In addition, we address the main limitations and challenges associated with SPR measurements and consider strategies for adapting the technique to meet the specific needs of antiviral research.

## 1. Introduction

Antiviral drug discovery is based on research, including both computational and empirical screening [1,2,3]. The first one is used to design molecules that can inhibit virus entry into cells, viral genetic material replication, or the processing of viral polyproteins. It is also used for the first selection of the most promising molecules. Although this strategy is quite fast and cost-effective, its results require experimental validation to ensure that effects predicted in silico are observed in vitro and in vivo. A wide variety of methods can be used to analyze the interaction between the putative drug and its molecular target, validate the putative drug’s safety in host cells, and, finally, assess efficacy in virus neutralization in vivo in animal models [1,2,3].

The designed small molecules or peptides predicted to prevent viral infection or inhibit viral propagation are first analyzed for their recognition and interactions with molecular targets. There are different strategies for identifying the most promising drugs: high-throughput screening (HTS), fragment-based drug discovery (FBDD), hit validation, or drug repurposing [4,5,6]. They rely on well-established methods for ligand–analyte interactions such as surface plasmon resonance (SPR) [7,8,9], Isothermal Titration Calorimetry (ITC) [10,11], MicroScale Thermophoresis (MST) [12,13], or Biolayer Interferometry (BLI) [11,14]. These methods are used for analysis of putative drugs against different viruses, e.g., severe acute respiratory syndrome coronavirus 2 (SARS-CoV-2) [9,12,13,15,16,17], human immunodeficiency virus 1 (HIV-1) [10,15], Hepatitis C virus (HCV) [12], herpes simplex virus type-1 (HSV-1) [18] and Influenza A virus [12,19,20].

Among the methods used to analyze biomolecular interactions, SPR is a gold-standard analytical tool. However, the specificity of interaction analysis using this technology makes SPR effective to varying degrees, depending on the approach chosen to identify the most active antiviral molecules. One of the biggest advantages of SPR over techniques such as MST or ITC is the determination of kinetic constants for putative drug interactions with viral or cellular targets [21,22]. SPR is an especially powerful technique when FBS is considered, because it allows detection of even very weak interactions, which constitutes the basis for this drug-discovery strategy. At the same time, it allows detection of binding of very small ligands (100–300 Da) [22]. These are undoubtedly advantages compared to a similar BLI technique, although SPR is not as high-throughput. The requirement of a small volume of label-free sample also often determines the choice of SPR over ITC (which usually requires higher concentrations of the molecules) [23] or MST (which requires fluorescent labeling of the ligand) [12,13]. On the other hand, both ITC and MST are solution-based methods that do not require ligand immobilization [11], which could be important when analyzing fragile or complexed viral proteins.

In this review, we will present the bases of SPR analysis with a focus on experimental design in antiviral drug discovery research and the utility of SPR in research targeting key steps of viral infection. We will highlight the limitations and challenges of SPR analysis and the potential for adapting the assay for antiviral research.

## 2. Designing SPR Experiments for Antiviral Drug Discovery

SPR analysis is based on a physical phenomenon that occurs at the interface between metal and dielectric material, when the polarized light hits the metal surface at an incident angle (resonance angle) and interacts with free conduction electrons, driving them to oscillate collectively [24,25]. These oscillating electrons are called plasmons. The incident light is absorbed by surface plasmons, leading to a decrease in reflected light intensity. The resonance angle is very sensitive to variations in the refractive index (RI) near the metal surface, and RI shifts are precisely measured, indicating changes in surface mass density and allowing the detection of interactions between biomolecules [24,26].

**Figure 1:**
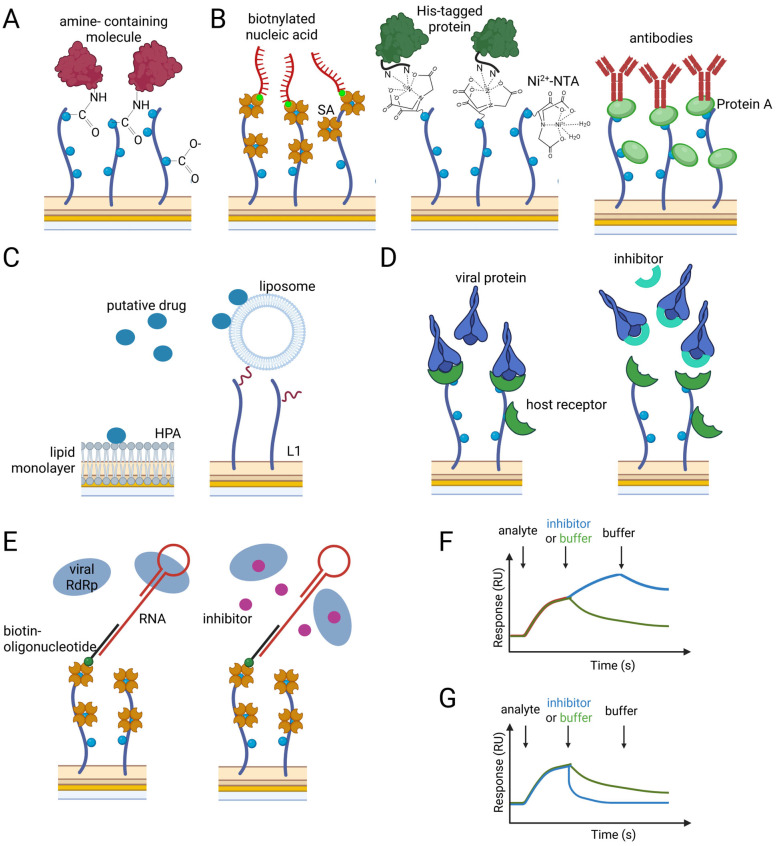
Schemes for sensor chip surfaces and ligand immobilization strategies in SPR for analysis of putative drugs. (**A**,**B**) An example of a sensor chip surface with dextran matrix for (**A**) amine-coupling immobilization of amine-containing molecule with available COO^−^ grups or (**B**) with already immobilized strepavidine, NTA-Ni^2+^ and protein A for immobilization of biotinylated, Hist-tag-containg molecules and antibodies, respectively. (**C**) Analysis of interaction between the putative drug and lipid monolayer or liposomes immobilized on HPA or L1 sensor chips, respectively. (**D**) Left panel: host receptor protein (e.g., ACE2) immobilized via amine groups and analysis of surface binding by viral protein alone (e.g., SARS-CoV-2 spike protein). Right panel: analysis of binding immobilized host receptor by viral protein preincubated with the tested inhibitor. An inhibitor that binds to the viral protein at the same interface as the host receptor blocks the receptor’s binding. (**E**) Left panel: analysis of viral RNA polymerase (RdRp) binding to immobilized RNA molecule alone. Right panel: analysis of viral RdRp binding after the preincubation with the putative inhibitor. (based on [31]). (**F**,**G**) Sensorgrams showing the response after injections of the analyte onto the surface with immobilized ligand, followed by injection of the inhibitor (blue) or buffer (green). The increase in signal after inhibitor injection indicates binding to the analyte (blue) (**F**). The decrease in signal after inhibitor injection, more significant than after buffer injection, indicates removal of the analyte from the complex with the ligand (blue) (**G**). Not drawn in scale.

It requires immobilizing the ligand on the sensor chip surface, covered with a thin layer (45–50 nm) of metal, usually gold. The metal surface is often covered with a layer of a polymer matrix (dextran, Polyethylene Glycol (PEG)), with functional groups that allow ligand immobilization directly or via capture molecules [27]. The most popular is the carboxymethylated (COO-) dextran matrix, which allows activation via amine-coupling chemistry (Figure 1A) [28]. This strategy is often used when the ligand is a protein, either a viral receptor-binding protein, a viral enzyme, or a host protein recognized by the virus, and the putative inhibitors of virus–host interaction are tested. Although the amine coupling method immobilizes the ligand stably on the surface and can be used with any molecule containing an amine group, it can also result in multiple ligand orientations when there are multiple such groups. Therefore, the same matrix is available covalently bound to streptavidin, allowing immobilization of biotinylated ligands, or to nitrilotriacetic acid (NTA), allowing capture of His-tagged proteins via Ni^2+^ or to Protein A/G capturing antibodies (Figure 1B). These immobilization methods ensure uniform orientation of the ligand molecules on the sensor chip surface [29,30,31]. The availability of such diverse surfaces allows the selection of the most convenient immobilization, especially for proteins. In case of nucleic acid–protein interaction analysis, immobilization of nucleic acid via the biotin–streptavidin interaction is the method of choice (Figure 1B).

This distinct strategy is used when the analysis of interactions with biomembranes is considered. It could involve the interaction between viral envelope proteins and cell membranes, which occurs during the fusion step of infection, as well as the investigation of fusion inhibitors and their interactions with cell membranes. For this purpose, hydrophobic association (HPA) sensor chips and lipid-capture sensor chips are used, which allow the capture of lipid vesicles (Figure 1C). On the HPA sensor surface, from an injected solution of small unilamellar vesicles, a lipid monolayer forms, and on lipid-capture sensor chips, due to long aliphatic anchors, intact liposomes can be captured [32]. The use of both sensor chips in research on inhibitors of virus–host cell fusion allows, based on calculated equilibrium constants, differentiation between surface adsorption of the inhibitor and its insertion into the hydrophobic core of the membrane [33].

Once the ligand molecule is immobilized, the binding of the analyte to the prepared surface can be measured. To ensure that the surface-attached ligand is active, the interaction with a known binding partner should be tested. It can be, for example, the analysis of viral–host protein interactions (e.g., the SARS-CoV-2 spike protein and the host receptor angiotensin-converting enzyme 2 (ACE2)) (Figure 1D, left panel), or the binding of a viral enzyme (e.g., a polymerase) to nucleic acid (Figure 1E, left panel). When putative drugs that inhibit such an interaction by blocking the binding interface are tested, their binding to the target molecule is first analyzed. These kinds of analyses are quite simple and involve successive injections of different concentrations of the analyte, which bind to the ligand, thereby changing the RI near the sensor chip surface. These changes are measured in real time as shifts in the resonance angle, which are converted into a signal in relative units (response units, RU) [34]. Since RU is proportional to the mass bound on the sensor surface (1 RU corresponds to 1 pg of bound molecule), the more analyte is bound, the higher the response that is detected. The efficient binding of a putative drug to its target does not always correlate with inhibition of the expected interaction. Therefore, the competition-binding assay can be performed. The increasing concentrations of a putative drug should be preincubated with the target molecule (the analyte) before injection. If the drug forms a complex with the analyte at the same interface as the immobilized ligand molecule, it should prevent the ligand–analyte interaction, and the more of the drug is preincubated, the less analyte can bind (Figure 1D,E, right panels). In a different approach, the injection of a putative drug can follow the injection of the analyte. In this case, the information about the interaction between the drug and the target molecule outside the ligand–analyte binding interface can be gained (when increase in the response after drug injection onto preformed ligand–analyte complex is observed) (Figure 1F), as well as information about potential removal of the analyte from the complex (when higher decrease in the response after drug injection onto preformed ligand–analyte complex is observed compared to the injection of the buffer) (Figure 1G).

In SPR analysis, the result from the functionalized surface is always compared with that from the reference one [35]. The reference surface can be either an uncoupled sensor surface (an empty one) or a surface with an immobilized variant of the ligand that does not interact with the analyte (e.g., a non-specific antibody or an inactive mutant). The signal from the reference surface is subtracted from the signal obtained for the analyte on the functionalized surface. The blank injection of buffer alone is also important, especially when the analyte is dissolved in a solvent such as DMSO, which significantly influences RI and thus the detected response. In such conditions, solvent correction should be performed. After each cycle of the analyte binding, the surface is regenerated. If the ligand is immobilized via a capture molecule, in the new cycle it should be immobilized again, unlike in covalent ligand immobilization. However, the regeneration step can affect the covalently immobilized ligand, so its activity should be monitored.

During SPR analysis, response is measured in real time, allowing the kinetic constants of the ligand–analyte interaction to be calculated [34]. From data collected during analyte injection onto the sensor chip surface with a fixed ligand, the association constant (k_on_) can be determined. Injection of the analyte is followed by flow of the running buffer over the sensor surface. Response detected during this step of the analysis indicates the rate of complex dissociation and allows calculation of the dissociation constant (k_off_). The kinetic constants are determined by fitting the data obtained during analysis to a kinetic binding model. The choice of an appropriate binding model is one of the crucial factors for obtaining the not-apparent but the most intrinsic constants. The most popular is the 1:1 Langmuir binding model, which is simple and assumes that each analyte molecule binds to a single ligand molecule, with no cooperativity or conformational changes [34,36]. Usually, it is sufficient for analyzing small-molecule interactions with the target protein, interactions of monovalent antibodies (Fab) with the antigen, and interactions of single-domain proteins [20,37,38]. When binding occurs in two steps with conformational rearrangements, the better model could be the two-state model [34,36]; it is often observed in ligand–receptor binding, peptide–protein binding, or compounds with long residence times [39,40]. For analytes with two known binding sites, such as antibodies, the bivalent analyte model should be the choice [41]. There is also a possibility that the heterogeneous ligand model will be used if the ligand partially denatures on the surface or is immobilized in two different orientations [34,36]. The kinetic constants are then calculated for two independent binding sites. From the response detected during association of various analyte concentrations, the steady-state affinity of the analyte to the ligand can also be calculated (equilibrium dissociation constant (K_D_)). Calculating affinity rather than k_on_ and k_off_ is recommended when the complex binds and dissociates rapidly, which is often the case with small-molecule analytes [42]. The selection of the appropriate binding model should always be based on biological context (e.g., whether the protein undergoes conformational changes or binds to two ligand molecules) and a critical inspection of the obtained data.

## 3. Targeting Steps of Viral Infection—Constraints and Adaptations in SPR Analysis

The process of viral infection can be divided into a few steps common to different viruses, despite their distinct structures, genetic material, modes of transmission, etc. [43]. First, the virus has to attach to and enter specific host cells; then, the viral genetic material should be released from the capsid, transported to the nucleus and replicated. To form new viral particles, viral genes must be expressed, leading to the synthesis and processing of viral proteins. When all viral components are present, they assemble into new virions and are released from the host cell, allowing the cycle to repeat. Antiviral strategies are based on inhibiting the most important steps of this cycle, e.g., adhesion and entry into cells, genome replication, and viral-polyproteins processing [44,45]. During the investigation of these steps, SPR can be successfully utilized if only the key constraints of this technique are considered.

### 3.1. Looking for Viral Entry Inhibitors

The best way to stop an infection is to act at the very first step, before the virus can spread through the body’s cells. To do this, molecules that inhibit the interaction between the viral envelope protein and its receptor on the host cell surface are designed. The molecule can bind either the viral protein or the host receptor in the interaction interface. Using SPR, these interactions are measured, as well as the decrease in complex formation when, prior to injection, the analyte is preincubated with the putative drug.

The typical targets when looking for viral entry inhibitors are quite large, complex (oligomeric) molecules, either when they are viral surface proteins (e.g., the SARS-CoV-2 virus’s spike protein (the S-glycoprotein) subunit S1; gp140 and gp120 of HIV-1) or host receptor (e.g., ACE2, CD4). The native proteins involved in viral entry are also membrane-associated; therefore, SPR analysis of full-length partners with proper quaternary structure is very difficult. Therefore, the research model is often simplified, and selected protein domains or monomeric proteins are analyzed. An example is the SARS-CoV-2 spike protein, where the S1 subunit is a trimer [46], but in SPR analysis, usually just its receptor-binding domain (RBD) is analyzed for interaction with ACE2 or neutralizing molecules [47,48,49,50]. Although this approach simplifies the analysis, it can yield apparent affinity and kinetic constants from the data because it does not account for multiple simultaneous interactions that occur in nature. Research comparing the interaction between the CD4 (presents on the surface of immune cells and constituates the primary receptor of HIV-1) and HIV-1 viral envelope proteins gp140 and gp120 purified as a stable trimer and a monomer, respectively, and investigating the neutralizing effect of selected antibodies on this interaction indicated significant differences resulting from the oligomeric state of the viral proteins [51]. The obtained results support the concept that gp140 trimers are better immunogens than gp120 monomers.

To support the determination of the most intrinsic interaction parameters, the availability of binding interfaces in both tested partners is crucial. Although in antiviral research the immobilization via amine group with the amine coupling method is also often applied (e.g., RBD-ACE2 interaction [47,49,52], CD4-gp120 [53]) to ensure the highest accessibility of binding site and to achieve a uniform orientation of the ligand, the immobilization via tag molecule is recommended (e.g., biotinylated ACE2, ACE2-Fc, RBD-Fc, His6-RBD [28,45,48,54]). Once the data are collected, the appropriate analysis is required. The default analysis usually applies the 1:1 Langmuir binding model. When considering the structural conditions mentioned above for the tested molecules, this model can provide an apparent description of the tested interaction. It fits well with simplified research systems, e.g., the use of RBD instead of the S1 protein [39]. However, as it was shown for S1-S2 stable spike protein interaction with ACE2, the application of a 1:1 model is burdened with error due to the failure to take into account minor conformational changes in the spike protein after initial receptor binding [39]. These limitations in applying the simplest model were overcome by using a two-state model, which resulted in a better fit, especially during the dissociation phase.

The application of a more complex model should also be considered in cases of investigation of antibody binding to neutralize the viral entry protein–host receptor interaction. Usually, the bivalent analyte model is applied when the antibodies are flowed over the immobilized target molecule. The 1:1 model is sufficient when antibodies are captured on the sensor chip surface, or monovalent nanobodies or Fab fragments are used as analytes. These kind of analyses were performed for investigation: nanobodies (e.g., H11 [48]) and antibodies (e.g., humanized mAbs MG1141A [30]) that should bind the S1 SARS-CoV-2 spike protein, preventing its ACE2 recognition; for investigation of binding antibodies, captured via immobilized human Fab binder or protein A, and HIV-1 viral proteins gp140 and gp120 as the analyte [52], or testing interaction between immobilized glycoprotein B (gB) of Epstein–Barr virus (EBV) and Fabs of neutralizing antibodies as analytes [55].

Unfortunately, correctly determining the kinetics of interaction at the stage of viral entry is not limited to selecting the appropriate immobilization method and binding model. Because the studied molecules are large, mass transfer and artifacts resulting from analyte rebinding can be significant limitations [34,56]. To increase the accessibility of the surface for the analyte, the low ligand immobilization level is applied (depending on the molecular weight of the ligand, usually 100–500 RU) and to remove the analyte from the surface immediately after dissociation, a high flow rate is set (up to 100 μL/min) [57,58,59].

These limitations do not occur when small antiviral molecules interact with their targets, whether viral envelope proteins or host receptors. The examples could be the interaction between immobilized HA and a small molecule; inhibiting membrane fusion of influenza virus and a host cell [60]; DCM205, compound-inhibiting interaction between the CD4 receptor and gp120 HIV-1 protein [54]; ficolin-1 binding GP glycoprotein of Ebola virus (EBOV) [61]; or mosquitoes salivary proteins AAEL000793, AAEL007420 and AAEL006347 binding the envelope protein of the Zika virus (ZIKV) [62] (Table 1). Small molecules binding to the ACE2 receptor were also analyzed. An example could be the analysis of interaction with ACE2, the derivatives of sartans (e.g., Losartan, Telmisartan, Olmesartan), which are known as Angiotensin II Receptor Blockers (ARBs) used for the treatment of hypertension and heart diseases [63]. These bis-alkylated imidazole derivatives bearing two anionic biphenyltetrazoles (bisartans) were designed to bind the ACE2 at its RBD interface [64,65]. In SPR analysis, it was shown that bisartan BV6 (bisalkylated) binds well to immobilized ACE2 protein and more strongly compared to losartan [52,65,66].

Although the disruption of direct contact between the viral protein and its host receptor is very important, and research is usually focused on this process, it is also worth noting that the inhibitory molecule can interact more or less specifically with the membrane in which the receptor is embedded. Studies using SPR by Cao and coworkers showed that peptide fusion inhibitors of HIV-1, sifuviride and enfuviride, might differ in efficacy due to higher local concentrations at the fusion site resulting from different interactions with rigid lipid areas [70].

During the viral entry into the host cell, the receptor is not always a protein. In many cases, the viral envelope protein (e.g., the RBD of the S1 spike protein [76], the envelope protein of Dengue virus [75], and the hemagglutinin (HA) of the influenza virus [77,78]) recognizes glycoconjugates present on the cell’s surface. Research conducted to identify this mechanism of viral entry by SPR usually utilizes the immobilization of tagged glycans (e.g., biotinylated glycans [78] or modified sialoglycopolymers [7] immobilized on a streptavidin-coated sensor chip immobilized surface). The interaction between glycoconjugates and viral protein can be eliminated by molecules structurally similar to the molecule of attachment. Usually it is heparine, but because it can cause some side effects such as bleeding [79,80], safer alternatives are being sought (e.g., the polyanionic compound Suramin behaving similarly to heparin, with a fast on-rate and an extremely slow off-rate during interaction with the envelope protein of the Dengue virus [75]; heparin mimetics interacting with RBD [67]). During analysis, a competition-binding assay is performed in which heparin can be immobilized on the sensor chip surface, and the viral protein, alone or in the presence of heparin mimetics, is flowed over it. Mimetics with affinity for viral proteins comparable to that of heparin are the most promising in preventing virus–host interaction [67]. Similarly, polyclonal antisera, monoclonal antibodies, or intravenous immunoglobulins can be analyzed [7], with respect to the constraints mentioned above.

### 3.2. SPR Analysis for Inhibition of Viral Enzymes

Although preventing virus entry into cells is the obvious target of antiviral drugs, research on new drug development also targets subsequent stages, such as expression of viral genes, proteolytic processing of viral polyprotein and replication of genetic material. The first two steps are required to obtain viral proteins, which can then act during viral genome replication, nuclear export, viral particle assembly, and their release. In SPR analysis, if the inhibition of polymerase activity or proteolytic processing of the polyprotein is investigated, the enzyme is usually immobilized on the sensor chip and viral enzyme inhibitors are flowed over the prepared surface (Table 1). To maintain enzyme activity, immobilization via a capture molecule (tag or antibody) is the most recommended method [57,58,59,60], although amine coupling is also often applied [69,72,81,82,83,84]. This kind of analysis was performed, for example, for the main protease of SARS-CoV-2 (3CL^pro^/M^pro^), which is critical for viral genome replication and thus an excellent target for drugs, especially since no homolog of M^pro^ has been identified in humans [85]. Analysis indicated efficient binding to and inhibitory effects on M^pro^ by natural products, including chebulagic acid (CHLA) isolated from *Terminalia chebula* [17], hydrolyzable tannins (ellagitannins and gallotannins) [83], and cannabinoids [84]. SPR also identified efficient viral protease inhibitors when drug repurposing was considered. The already approved and investigational drugs were screened against ZIKV, and from them, temoporfin was shown to bind the ZIKV protease NS3 domain the strongest [74]. Also, tests of compounds with inhibitory activity against the NS3/NS4A protease of HCV identified those that bind the NS2B-NS3 protease of ZIKV as well [81]. This strategy with enzyme immobilization was also presented in the study of Lo and coworkers, which allowed for identification of compounds from a library of over two hundred, targeting the C-terminal domain of PA subunit (PAC) of RNA-dependent RNA polymerase (RdRp) of influenza virus [68], or in other studies for inhibitors of NS5B polymerase of HCV [71,86].

The strategy of immobilizing a viral protein and injecting a putative inhibitor was also applied to other viral proteins required for genome replication. An example is VP30, the protein critical for EBOV RNA synthesis [87,88]. Small molecules, embelin and Kobe2602, selected from a library of 8004 compounds, were tested for their inhibitory activity to VP30 with SPR in different configurations. They were either immobilized on the sensor chip surface and tested for VP30 binding at the interface, interacting with other proteins, or flowed over the surface with immobilized or captured VP30 [73]. Their binding and inhibitory effects were confirmed. Another example is the analysis of A9, a ligustrazine derivative, and its interaction with an immobilized His-tagged NP protein from the influenza virus [89]. This interaction was shown to inhibit the nuclear export of viral ribonucleoprotein (vRNP).

The level of enzyme immobilization has to be sufficient to detect the signal from the binding of inhibitors, which are usually very small molecules (200–800Da). Due to the size of the analyte and, consequently, low detected response, this research system is characterized by high sensitivity to bulk refractive index changes [90]. Therefore, the buffer has to be properly selected, and if an organic solvent such as DMSO is used, a solvent correction is mandatory [91]. Reversing the research system and immobilizing small molecules could reduce the limitations imposed by the small molecular weight of inhibitors, and keeping the enzyme in solution could increase its activity; however, the use of small analytes allows the application of the 1:1 binding model for analysis. Analytes with complex structures, such as oligomeric, multi-domain proteins (e.g., proteases and polymerases), may make it difficult to select the appropriate binding model. On the other hand, the binding of small molecules can occur too rapidly to allow reliable determination of kinetic constants. In this case, only the affinity at equilibrium can be determined [82].

Since viral polymerase activity depends on binding to nucleic acid, which can be easily immobilized if biotinylated, allowing the enzyme to remain in solution, this configuration can be successfully used for screening polymerase inhibitors. It was proposed by Mravinec and coauthors, who immobilized an RNA molecule composed of annealed oligonucleotides, one biotinylated and the other complementary, which interacted specifically with RdRp [31]. As an analyte, RdRp alone or after incubation with antiviral drug (ribavirin, favipiravir, sofosbuvir and suramin) was used. The comparison of RdRp binding to RNA before and after incubation with drugs indicates that suramin precluded interaction and even displaced RdRp from RNA when it was flowed over the surface with preformed RNA-RdRp complexes [31].

### 3.3. Assembly and Budding Inhibitors

The latest steps of viral infection, which can be targeted by antiviral drugs, are the assembly of viral particles and their release from cells. Since these processes involve multicomponent and multivalent interactions and are linked to lipid membrane interactions [84,85,86], SPR is not the most popular technique for assessing them. Usually, the binding of monomeric proteins instead of oligomers is analyzed. However, as shown for the HIV-1 capsid protein (CA), the oligomeric state of the target during SPR analysis may not only influence the determined kinetic parameters but also the occurrence of binding [92]. During the analysis, the CA protein was immobilized as a monomer, a hexamer, or just as its N-terminal domain (CA-NTA), and binding of the selected inhibitors (H22 and PF-74) was observed only with the hexamer. As for other small-molecule inhibitors, also in this case, the proper calculation of kinetic constants was difficult, and the obtained data were apparent compared to those obtained with other solution-based methods like ITC [92]. Therefore, only the equilibrium dissociation constant was calculated. Similarly, kinetic constants were not determined for CA inhibitors identified in other studies [93].

The use of antibodies as neutralizing molecules may overcome the constraints imposed by characteristic small-molecule binding, but in this case, mass transfer should be avoided. Neutralizing antibodies that inhibit this stage of viral infection were investigated, for example, for EBOV [94] and influenza virus [20,59,95]. In studies on EBOV, the viral protein VP40 was immobilized via amine coupling and tested for recognition by anti-VP40 antibodies. In the same assay, the selected antibodies were tested for recognition of other viral proteins to indicate possible off-targets and those that bound specifically to VP40 were identified [94]. In research on the influenza virus, the second major protein, neuraminidase (NA), which facilitates the release of viral particles from infected cells by cleaving sialic acid [96], was captured via a polyclonal anti-Avi antibody and the Avi-tag fused with NA [95] or via a modified known NA inhibitor (HAD-zanamivir) [20,59]. These strategies allow analyzing, on the surface of a single sensor chip, the interaction between different NA variants, e.g., those with diverse glycosylation [95] or wild-type and point substitution variants [20,59]. Research on capturing different NA variants on the sensor chip surface shows that this strategy could be used to rapidly screen newly emerging NA variants for their sensitivity to antiviral drugs. Even if the binding parameters are apparent, their comparison across different target variants allows the selection of the most prominent molecules for investigation with other solid- and cell-based techniques.

## 4. Limitations, Challenges and Future Perspectives

Although the SPR technique is well established in antiviral drug discovery, it is not without limitations (Table 2) and it should be used in combination with other methods, allowing for resolution of the complex structure and in vivo effects.

SPR is considered a label-free method because detection does not require labeling, and immobilization on the sensor chip surface can be achieved via groups present in the molecules, e.g., the amine group of proteins. However, this type of immobilization can result in a different particle arrangement on the surface, which can influence interaction. Immobilization via a capture molecule bypasses these limitations but requires either tagging the ligand (e.g., biotin, His-tag) or the availability of capturing antibodies. Nevertheless, even if the molecules are deposited equally on the surface, it is necessary to ensure that the immobilization does not mask the interaction interface and that the interface remains accessible to the analyte. As shown, the oligomeric state may also influence binding, and purification of stable oligomers is required to achieve efficient complex formation [92,97]. It is also worth noting that many viral proteins involved in virus entry, as well as those connected with virus release, are embedded in membranes, and recreating native conditions (e.g., proper protein conformation) during SPR analysis can be complex and challenging, even when a lipid bilayer is used to capture the protein. When analyzing proteins with complex glycosylation, it is even more challenging to produce them homogeneously and thus achieve efficient, reproducible results. The system used for protein production can influence posttranslational modifications and, consequently, binding kinetics, as observed for the SARS-CoV-2 S1 protein produced in different cells: baculovirus-insect, Chinese hamster ovarian, and human embryonic kidney [98]. The glycosylation of viral proteins can also influence the background, since glycan groups can interact nonspecifically with the reference surface [99].

In SPR analysis, the standard 1:1 binding model is typically used to describe interaction kinetics, assuming a simple, single-step binding. Application of the simplest equations is usually sufficient for small monovalent analytes and single-domain proteins. This model also usually works when enzyme (e.g., polymerase or protease) active-site inhibitors are tested. However, viral–host interactions often involve multiple binding sites, are cooperative, or involve conformational changes, making simple models insufficient. Sevenich and coauthors showed, using the example of the SARS-CoV-2 spike protein and human ACE2 interaction, that choosing a binding model other than the 1:1 results in better fitting of experimental data [39]. Applying the model assuming a conformational change after the initial binding (the two-state model) better correlates with the data, especially when complex dissociation was measured over a longer time. The observed slower than expected dissociation usually indicates that multiple bonds have to break simultaneously, and the parameters calculated with a 1:1 binding model are apparent [34]. Thus, proper experimental design and data analysis to avoid artifacts are critical. If it is known that bivalent analytes or heterogeneous ligands are tested, then the dedicated model should be used. The solution could also involve inverting the orientation and use of a multivalent molecule as a ligand rather than an analyte. The ligand could also be immobilized at different densities, and the slower off-rate at the higher density might indicate multivalent rebinding [100]. Nevertheless, obtaining data from other methods (e.g., ITC, MST, cryo-electron microscopy, X-ray crystallography) should always be considered to get the most relevant information about the potential multivalency, activity and mechanism of action of the putative drug. On the other hand, even when complex formation is best characterized in vitro, it will not always reflect the actual cellular effect. Usually, data obtained from techniques such as SPR correlate well with antiviral efficacy for inhibitors that directly block the catalytic center of the well-characterized target. Also, the molecule’s long residence time is a good predictor of its inhibitory activity. However, very often in antiviral research, in solid-based techniques, isolated proteins’ domains or monomers are used, which do not reflect the conformational state observed in nature (the multiprotein complexes or oligomers, embedded in membranes). Above all, it shouldn’t be forgotten that SPR does not determine putative drug uptake, efflux, metabolic stability, cytotoxicity, or, finally, antiviral activity; thus, functional assays must be conducted because target engagement does not always reflect antiviral efficacy. In the work of Dierynck and coworkers, variants of HIV protease, derived from clinical isolates and harboring protease inhibitor resistance-associated mutations, were immobilized and analyzed for interaction with various protease inhibitors [69]. It was shown that many of them were bound to proteases containing substitutions in crucial residues, resulting in lower affinity and, consequently, lower antiviral activity. Darunavir was an exception, and a nearly 1000-fold decrease in binding affinity did not change its activity against HIV protease variants [69].

To improve the identification of putative antiviral molecules and their better characterization, machine learning (ML) can be applied [2,101,102,103]. It can be considered for anything from experimental design, through parameter setting analysis, to data processing [2,103,104,105,106]. The application of artificial intelligence, from the very first step of designing molecules to in silico validation, can significantly increase the success rate of experimental results. A study by Liu and coauthors used an integrated computational workflow (including large-scale virtual screening of over 10 million compounds, molecular mechanics/generalized born surface area (MM-GBSA) calculations, and molecular dynamics (MD) simulations) to identify novel influenza A nucleoprotein inhibitors, leading to the experimental validation of 16 candidates, of which 3 compounds showed the strongest target engagement in SPR assays [107]. Also, in research on other viruses, the initial in silico selection of antiviral molecules for further in vitro (with SPR [52,66,108,109,110]) and in vivo experiments was conducted [2,111,112,113,114], which limits the number of analyzed molecules. ML was also successfully applied to predict effective antiviral combinations of known antifvirals, confirmed by experiments (i.a., SPR) demonstrating increased binding affinity and viral suppression [115].

Regarding SPR analysis, ML is likewise particularly useful during the data processing step because it enables faster sensorgram classification based on binding models, aggregation, mass-transport limitations, or bulk artifacts [116]. It also provides better kinetic parameter calculations when the standard binding model is not accurate. Its potential can also be used to predict off-target interactions, which are missed entirely when binary interactions (viral protein–antiviral molecule) are measured by SPR [117]. The only limitation to applying ML to SPR data processing is the availability of a large training dataset. ML can also be utilized to design improved sensors that will provide higher sensitivity, selectivity, stability, and lower detection limits. The ML-designed improved sensors are now being tested, in which a classical gold or silver layer is replaced or modified with novel plasmonic materials (e.g., graphene) [24,118,119,120]. Therefore, leveraging the potential of artificial intelligence can accelerate research and help design drugs that target specific targets.

## Figures and Tables

**Table 1:** Examples of SPR analysis in research concerning antiviral molecules.

Virus	Ligand Immobilized	Analyte	Reference
SARS-CoV-2	main protease	chebulagic acid (CHLA)	[17]
	RNA	RNA-dependent RNA polymerase ±RNA polymerase inhibitors	[31]
	heparin	spike trimer glycoproteins ±heparin mimetics	[67]
	RBD	Abs	[47]
	RBD	nanobodies	[48]
	mAbs	S-protein	[30]
	ACE2	bisartans	[52]
	RBDs	ACE2	[49]
Influenza virus	PAC (C-terminal domain of RNA polymerase)	library of 165 compounds	[68]
	Viral particles	Fabs of mAbs	[29]
	HAD-zanamivir	Neuraminidase ± NA-inhibitor	[20]
HIV	goat anti-human Abs capturing anti-gp41 and gp120 mAbs	oligomeric gp140 ±DCM205 (small-molecule inhibitor)	[54]
	HIV-1 protease	Protease inhibitors (amprenavir, atazanavir, darunavir, lopinavir, and tipranavir)	[69]
	17b, sCD4 and b12	gp120 protein	[53]
	SUVs	peptides (sifuvirtide and enfuvirtide)	[70]
HCV	NS5B polymerase variants	small-molecule inhibitors	[71]
	the nonstructural protein 3 (NS3) variants	NS3 protease inhibitors	[72]
EBOV	glycoprotein (GP) variants	ficolins	[61]
	VP30 protein	Embelin, Kobe2602, Kobe0065, 8-gingerol	[73]
ZIKV	NS3 protease subunit	Protease inhibitors (temoporfin, niclosamide, nitazoxanide)	[74]
	ZIKV-E protein	*Aedes aegypti* salivary proteins	[62]
Dengue virus	envelope protein	suramin	[75]
HSV	glycoprotein D (gD)	sulfated glycans	[18]

**Table 2:** Constraints and SPR assay adaptations depending on the type of analyzed molecules.

Molecules	Viral Infection Step	Constraint	Assay Adaptation
Viral surface proteins Host receptors	Virus attachment and entry	Mass-transport limitation	Low ligand immobilization; fast flow rate
		Rebinding artifacts	Fast flow rate; low ligand density
		Conformational changes	Usage of the two-state binding model
Enzymes (polymerase, protease)	Genes expression Polyprotein processing Genome replication	Need for cofactor or nucleic acid	Immobilization of nucleic acid, addition of a cofactor in a buffer
		Structural complexity	Usage of protein domain
		Weak or transient inhibitor binding	Competition or displacement assays
Neutralizing antibodies	Blocking virus attachment	Mass-transport limitation	Low ligand immobilization; fast flow rate
		Bivalent binding	As an analyte—usage of the bivalent binding model; as a ligand—usage of the 1:1 binding model
Small molecule inhibitors	Inhibition of viral entry and enzyme activity	Low signal	Higher ligand density
		Solvent artifacts	Solvent correction
		Fast kinetics	Steady-state affinity calculation
		Non-specific binding	Usage of the reference surface and detergent

## Data Availability

No new data were created or analyzed in this study.

## Abbreviations

The following abbreviations are used in this manuscript:

SPR	Surface Plasmon Resonance
ITC	Isothermal Titration Calorimetry
MST	MicroScale Thermophoresis
BLI	Biolayer Interferometry
SARS-CoV-2	The severe acute respiratory syndrome coronavirus 2
HIV-1	The human immunodeficiency virus 1
HCV	Hepatitis C virus (HCV)
HSV-1	herpes simplex virus type-1
EBOV	Ebola virus
ZIKV	Zika virus
NHS	N-hydroxysuccinimide
EDC	1-ethyl-3-(3-dimethylaminopropyl)carbodiimide
ACE2	The receptor angiotensin-converting enzyme 2
RDB	The receptor-binding domain (RBD)
HPA	hydrophobic association
DMSO	dimethyl sulfoxide
ML	machine learning
MD	molecular dynamics
